# Next generation sequencing yields the complete mitogenome of Smith’s zokor (*Eospalax smithii*)

**DOI:** 10.1080/23802359.2020.1765211

**Published:** 2020-05-18

**Authors:** Zhenyuan Cai, Jingjie Zhang, Penghai Qiao, Wen Qing, Tongzuo Zhang

**Affiliations:** a Key Laboratory of Adaptation and Evolution of Plateau Biota, Northwest Institute of Plateau Biology, Chinese Academy of Sciences, Xining, China;; b QinghaiProvincial Key Laboratory of Animal Ecological Genomics, Xining, China;; c College of Life Sciences, University of Chinese Academy of Sciences, Beijing, China;; d Institute of Laboratory Animal Science, Guizhou University of Traditional Chinese Medicine, Guiyang, China

**Keywords:** Smith’s zokor, *Eospalax smithii*, mitochondrial genome, next generation sequencing

## Abstract

Sequencing analysis of mitochondrial genomes is useful for understanding the genome structures. In this study, complete mitochondrial genomes of the *Eospalax smithii* was obtained by using next generation sequencing method. The complete mitogenome of *E. smithii* was 16,350 bp long, containing 13 protein-coding genes (PCGs), 2 ribosomal RNA (rRNA) genes, 22 transfer RNA (tRNA) genes, and 1 non-coding control region (D-loop). The overall base composition of the heavy strand is A (33.65%), C (23.80%), T (30.31%), and G (12.24%). The base compositions present highly biased toward A + T nucleotides. The result of phylogenetic analysis showed the five *Eospalax* species formed a monophyly with the high bootstrap value and as a sister group of the genus *Myospalax*. This is the first report of the complete mitochondrial genomes of *E. smithii* and the mitogenome is potentially important for evolutionary biology, population genetics, and species diagnosis studies of the Mysopalacinae.

The Smith’s zokor, *Eospalax smithii*, is a typical subterranean rodent species and endemic in China (Fan and Shi 1982) belonging to subfamily Myospalacinae, family Spalacidae (Norris et al. 2004).

In this study, we successfully sequenced the first complete mitogenome of *E. smithii* by next-generation sequencing. The sample of *E. smithii* was collected from Gansu, China (N34.50°, E103.85°). Voucher specimens (No. MX-14) are stored at Key Laboratory of Adaptation and Evolution of Plateau Biota, Northwest Institute of Plateau Biology, Chinese Academy of Sciences. Total genomic DNA was extracted from muscle using DNeasy Tissue Kit (QIAGEN). An Illumina library was generated from genomic DNA and sequenced on an Illumina Hiseq 2500 platform. The complete mitogenome sequence of *E. smithii* was assembled, annotated, and analyzed.

The accurate annotated mitochondrial genome sequence of *E. smithii* (GenBank accession number MH891800) is a circular double-strand DNA molecule of 16,350 bp, containing 13 protein-coding genes (PCGs), 2 ribosomal RNA genes (rRNAs), 22 transfer RNA genes (tRNAs), and 1 non-coding region. The arrangement of the multiple genes is similar in line with other Myospalacinae species (Liu et al. 2011; Su et al. 2013; Li et al. 2016; Yuan et al. 2016; Cai et al. 2019). The overall base composition of the heavy strand is 33.65% A, 23.80% C, 30.31% T, and 12.24% G. The base compositions present highly biased toward A + T nucleotides. The heavy strand (H-strand) encodes 2 rRNA genes, 12 PCGs, and 14 tRNA genes, the *ND6* gene, and 8 other tRNA genes are encoded on the L-strand.

Nine of all 13 PCGs initiated with an ATG start codon except for *ND1*, *ND2*, *ND3* and *ND5*, which began with GTG, ATT, ATA and ATT start codon, respectively. Nine of the 13PCGs use TAA as the stop codon. The *ND1*, *ND2* and *CO1* are stopped with TAG, and *ND4* end with incomplete stop codon T. The 12S and 16S ribosomal RNA genes are 941 and 1569 bp long, respectively. The tRNA genes vary from 60 to 75 bp in length and employ the anticodons typical of vertebrate mt-tRNAs. Twenty-one tRNAs had a typical secondary structure (cloverleaf structure) except the *tRNA-Ser* (GCT), whose complete dihydrouridine arm was lacking. A total of 9 mismatched base pairs (5 U–U pairs, 2 A–A pairs, 1 A–G pairs, and 1 C–A pairs) were detected in tRNAs secondary structures. In our study, the non-coding control region of the *E. smithii* mtDNA is 933 bp long.

Phylogenetic relationships were inferred from available mitogenomes of 14 species of Spalacidae using Maximum Likelihood (ML) method with 13 PCGs and 2 rRNAs. The results of phylogenetic analysis displayed that *E. smithii* formed a clade with other species belonging to the *Eospalax* genus, the monophylies of genera *Eospalax* and *Myospalax* was confirmed and they formed sister group, and Rhizomyinae was a basal clade relative to others within Spalacidae (Figure 1). This mitogenome sequence of *E. smithii* would help in evolutionary biology, population genetics, and species diagnosis studies of the Mysopalacinae.

**Figure 1.**
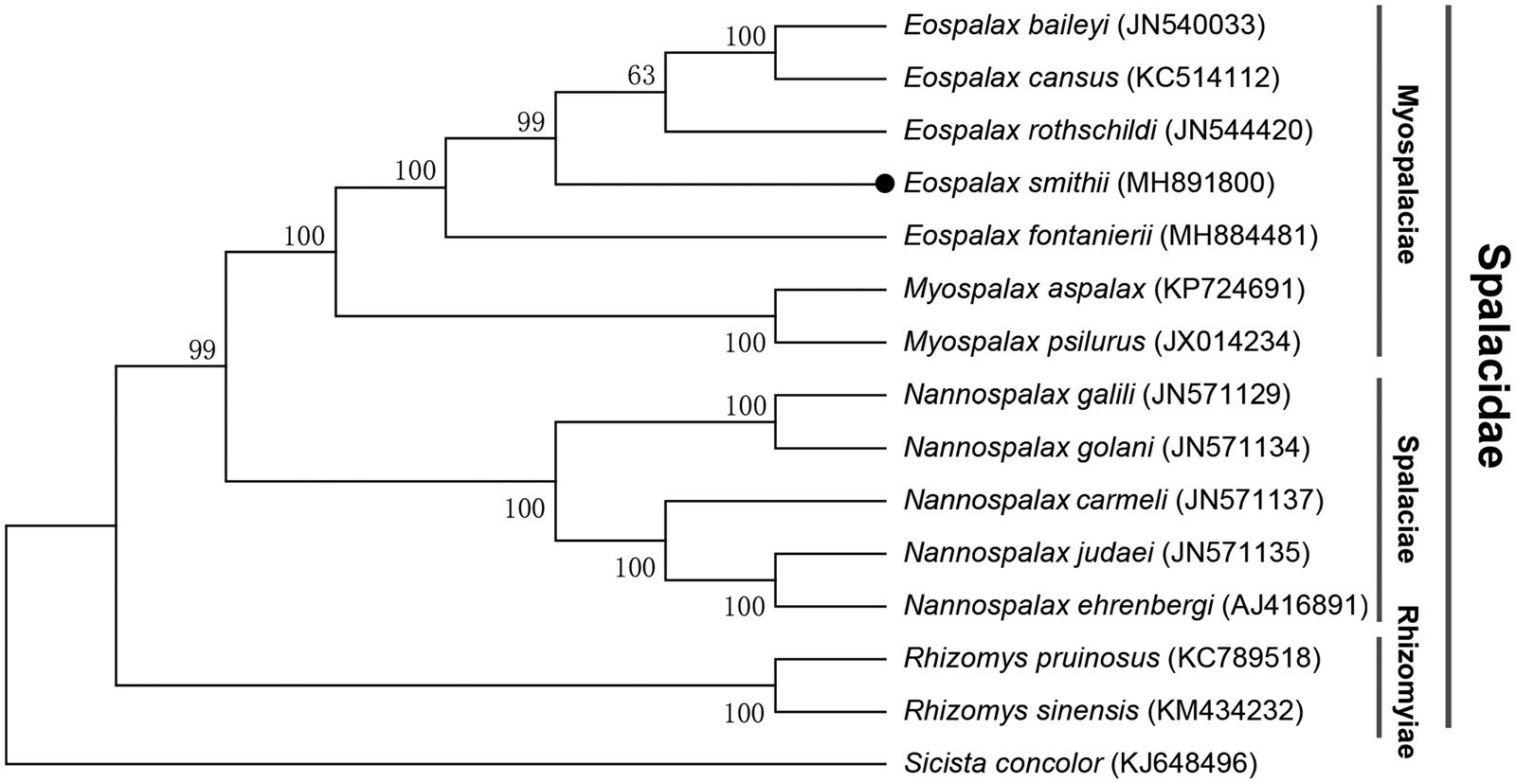
Maximum-likelihood (ML) phylogenetic tree of *Eospalax smithii* and the other 13 species of Spalacidae using *Sicista concolor* as an outgroup. The number around each node indicates the ML bootstrap support values.

## Data Availability

The data that support the findings of this study are openly available in NCBI Sequence Read Archive (SRA) at https://trace.ncbi.nlm.nih.gov/Traces/sra/sra.cgi?view=search_obj, accession number SRR11637901.
